# Sociodemographic and Geographic Influences of Mental Health Literacy: A Cross-Sectional Survey Among Community Health Clinic Attendees in Tshwane, South Africa

**DOI:** 10.3390/ijerph23020228

**Published:** 2026-02-11

**Authors:** Oratilwe Penwell Mokoena, Eric Maimela, Dumisile Priscilla Madlala, Thembelihle Sam Ntuli

**Affiliations:** 1 Department of Mathematics and Statistics, Faculty of Science, Tshwane University of Technology, Pretoria 0183, South Africa; 2 Faculty of Medicine and Health Sciences, Walter Sisulu University, Butterworth 4960, South Africa; emaimela@wsu.ac.za; 3 Department of Health, Faculty of Science, Tshwane District Mental Health Services, Tshwane 0001, South Africa; dumipsm@gmail.com; 4 Department of Statistical Sciences, School of Science and Technology, Sefako Makgatho Health Sciences University, Medunsa 0204, South Africa; sam.ntuli@smu.ac.za

**Keywords:** logistic regression, mental disorders, mental health literacy

## Abstract

**Background:** Mental health literacy remains low in South Africa, particularly in nonurban settings. This study aims to determine the sociodemographic and geographic influences of mental health literacy among community health clinic attendees. **Methods:** This study used secondary data which adopted a cross-sectional study design and was conducted between November 2019 and January 2020. A total of 385 participants were recruited through convenience sampling, with approximately 77 individuals per clinic across five sites. A two-part questionnaire was used, where part A included demographic information and part B consisted of the three fictive clinical case studies which measured the participants’ mental health literacy. The participants’ responses regarding disorder recognition and perceived causes were analyzed via Pearson’s chi-square tests. Using three fictive cases with clinical pictures indicative of mental depressive disorder, schizophrenia and general anxiety disorder, the following were assessed: (1) what type of illness do you think the person is suffering from, and (2) what do you think causes the persons’ suffering? To identify predictors of recognition and perceived causes, hierarchical logistic regression was performed. Statistical significance was set at *p* < 0.05. All analyses were conducted via STATA version 18.1 (StataCorp, College Station, TX, USA). **Results:** The mean age of the study participants was 37.39 ± 11.14 years (range: 13–80). Factors such as geographic location, gender and level of education were significant predictors of recognition. Participants attending urban clinics were more likely to correctly identify correct mental disorders than those attending township clinics were [OR = 0.32; 95% CI: (0.11, 0.93); Wald $\chi^{2}$(1): 4.3681; *p* value = 0.036]. For correct causes, significant predictors included gender, education level, and geographic location. Urban clinic attendees were significantly more accurate at identifying the correct cause of mental disorders than township attendees [OR = 0.42; 95% CI: (0.21, 0.83); Wald $\chi^{2}$(1): 6.1504; *p* value = 0.013]. **Conclusions:** Mental health literacy in Tshwane community healthcare clinics reflects deep-rooted sociodemographic and geographic inequalities. Strengthening township clinic capacity, integrating culturally relevant health education, and prioritizing gender-sensitive outreach are essential to improve the recognition and understanding of mental disorders in underserved communities.

## 1. Introduction

Mental health literacy (MHL) is increasingly recognized as a critical component of public health, yet it remains unevenly distributed across communities. In many low-resource settings, including parts of Tshwane, South Africa, individuals face significant barriers to understanding, recognizing, and responding to mental health challenges [1,2,3,4]. These barriers are shaped by a complex interplay of socioeconomic conditions, cultural beliefs, and systemic inequalities [5,6,7]. Without adequate MHL, communities are less likely to seek timely care and perpetuate cycles of stigma, misdiagnosis, and untreated illness. Addressing this concern is essential for improving mental health outcomes and strengthening the resilience of public health systems. In South Africa, the legacy of apartheid educational inequalities continues to shape access to healthcare and education, particularly in township communities [8,9,10]. While mental health disorders are increasingly recognized globally, many South Africans, especially those in historically marginalized settings, lack the ability to recognize and understand mental disorders. This gap in MHL not only reflects systemic inequalities in public health infrastructure investment but also perpetuates stigma, particularly among men and delayed treatment in the communities most in need. This study focuses on three fictive cases with clinical pictures indicative of mental depressive disorder, schizophrenia, and general anxiety disorder to measure MHL at a community level. These three mental disorders were selected due to their commonality in the South African setting [11]. Despite the global prevalence of schizophrenia being low, it is nonetheless a relatively common mental disorder [12]. Moreover, symptoms of schizophrenia seem to be more likely identified as belonging to a mental disorder, especially in African countries [13,14].

The City of Tshwane, like the broader South Africa, faces a significant mental health burden, characterized by high prevalence rates of disorders such as anxiety and depression and inequalities in service accessibility [15,16,17]. The national mental health context indicates that on average 16% of the South African population is affected by mental health issues, with up to 44% of young adults reportedly living with some form of mental health disorder [15,18]. A major health crisis facing the South African health system is the treatment gap, with reports indicating that nearly 75% of people with mental health disorders in South Africa do not have access to any form of mental healthcare [16,17]. This is due to a shortage of professionals, limited resources, and uneven distribution of services as a consequence of department of health budget cuts [17,19]. In response, the City of Tshwane and provincial government have integrated mental health services into its primary healthcare system and provide various public and private support options [20].

There is wide variation in the prevalence of mental health disorders globally; in Hunan Province, China, the prevalence of MHL ranges from 18.7% to 94.15% [21]. Another study in Foshan, China, revealed a significantly lower prevalence of 8.46%, with notable gender disparities, particularly in males having lower MHL than females did [4]. Several studies in Africa and South Africa have revealed disproportionate prevalence of MHL, in Nigeria, Aluh et al. [22] reported low levels of MHL, particularly that 4.8% of the participants correctly identified depression as a mental health disorder. Another study by Aluh et al. [23] further revealed that 12.1% of the students correctly identified and labeled the schizophrenia as a mental health disorder. In southwestern Ethiopia, 28% of the participants had poor knowledge of mental disorders [1]. Another study in Ethiopia by Mideska et al. [24] revealed high MHL levels among traditional healers in Jimma town. In South Africa, Zita [25] reported that 39.1% of participants were able to identify the major symptoms of common mental health disorders about the excessive use of substances. Madlala et al. [26] in South Africa revealed that participants displayed varied prevalence of common mental health disorders, 80.71% for mental depressive disorder, 63.83% for substance induced psychotic disorder, and 67.91% for social anxiety disorder. Matsoele and Tadi [27] further reported varied prevalence of MHL among black South African communities, depression 66.3%, schizophrenia 65%, and alcohol use disorder 85%. This varied prevalence highlights the sociodemographic divide in mental health literacy within the South African health system. Another study in KwaZulu Natal (KZN) by Nzama [28] revealed that 58.4% of participants in Umlazi, and 41.6% in KwaMashu correctly identified stress or depression as a mental disorder. In Johannesburg, Smit and Marais [29] reported high MHL levels among healthcare workers with a median score (inter quartile range) of 28 (25–30) for participants who correctly identified mental health disorders, and a median score of 6 (6–7) for participants who identified the correct cause of mental disorders. In Jordan, 71.1% of the study participants reported average MHL [2]. In Iran, Solhi et al. reported that 52.2% of study participants reported moderate levels of MHL [3]. This varied prevalence is associated with several covariates, such as sociodemographic, economic, and environmental factors [1,2,3,4,30].

In the context of MHL, the literature predominantly uses standardized questionnaires to assess public knowledge, attitudes, and practices related to mental disorders [1,2,3,4,31,32,33]. While these instruments provide valuable insights, they often lack contextual richness and fail to capture the subtle recognition of mental health conditions across diverse sociocultural settings. Notably, few studies have incorporated clinical imagery or vignettes to evaluate the recognition and causal attribution of common mental disorders, particularly in low-resource or heterogeneous environments. This study aims to determine the sociodemographic and geographic influences of mental health literacy among community health clinic attendees. By employing hierarchical logistic regression (HLR), this study further elucidates the sociodemographic, economic, and environmental covariates of disorder recognition and causal beliefs, offering a methodologically rigorous and contextually grounded contribution to the sequelae of MHL after the pandemic.

### Theoretical Background

Mental health literacy (MHL) is commonly defined as the knowledge and beliefs about mental disorders that aid their recognition, management, or prevention. Jorm’s framework identifies six components: (a) recognition of specific disorders or types of psychological distress, (b) knowledge and beliefs about risk factors and causes, (c) knowledge and beliefs about self-help interventions, (d) knowledge and beliefs about professional help available, (e) attitudes facilitating help-seeking and recognition, and (f) knowledge of how to seek mental health information. In this study, we focus on two core dimensions disorder recognition of disorders and knowledge of causes because these are pivotal gateways to timely help-seeking and evidence-based care [34,35]. MHL is embedded within broader health literacy and social determinants frameworks, which emphasize that literacy is shaped by education, sociodemographic and economic status, and cultural context [5,6,7]. These determinants significantly influence disparities in mental health outcomes and access to care.

A consistent theme in the literature is that women consistently outperform men on MHL, particularly in recognition of common disorders and identifying correct causes. Proposed mechanisms include gendered socialization around emotional expression, differing help-seeking norms, and the burden of gender-based violence that heightens contact with mental health information and services among women [4,33,36,37]. In contrast, masculinity norms that give toughness and self-reliance often suppress men’s help-seeking and reduce engagement with mental health knowledge, thus lowering MHL among men. These dynamics supports the inclusion of gender as a key determinant in our models [38,39,40].

South Africa’s historical education inequalities, rooted in apartheid-era policies (e.g., the Bantu Education Act of 1953), have left enduring legacies: under-resourced schools, de facto segregation, and uneven educational attainment [41,42,43]. These structural inequities translate into gaps in MHL, particularly for populations served by township clinics. Accordingly, level of education is a theoretically motivated determinant of both recognition and cause of mental health disorders. Furthermore, urbanicity is theorized to increase exposure to health information, specialist services, and training opportunities for frontline workers, potentially enhancing community-level MHL [31,32,44,45]. In contrast, township settings often face among others, resource constraints, and fewer training opportunities. These inequalities justify geographic location as a structural predictor of MHL components in our study.

Vignette-based instruments mapped to DSM-5 criteria are widely used to measure public knowledge of recognition of mental disorders and correct causes, and they are particularly valuable in heterogeneous sociocultural contexts because they provide concrete symptom narratives [33,34,46]. This study uses three fictive clinical vignettes (depressive disorder, schizophrenia, generalized anxiety disorder) and asks respondents (i) identify whether the case depicts a mental illness and (ii) select the most accurate causal explanation (biological/genetic/psychological) versus non-evidence-based causes (e.g., punishment/bewitchment). This operationalization directly targets knowledge of recognition and causes of mental disorders as defined by the MHL framework.

## 2. Materials and Methods

### 2.1. Study Design and Setting

This study uses secondary data from a quantitative, cross-sectional, descriptive study which was carried out between November 2019 and January 2020. The study was performed in region 1 of Tshwane, in the Gauteng Province of South Africa. Gauteng is one of the nine provinces in South Africa. Tshwane is one of five metropolitan municipalities in Gauteng Province, which is further divided into seven demographic regions. The city has a population of about 2.2 million, with region 1 having a population of approximately 811,570. Moreover, City of Tshwane has about 61 clinics that serve approximately 250 people daily in each clinic. The study which provided the data was performed at five of these clinics, namely, the Tlamelong Clinic, Kgabo Clinic, Phedesong 4 Clinic, Soshanguve Clinic 3, and Pretoria North Clinic. The latter is the only urban clinic. The clinics were randomly selected.

### 2.2. Sample Size, Technique, Inclusion, and Exclusion Criteria

The study which provided the data included a total of 385 participants recruited via convenience sampling. This total sample included 77 participants per clinic. The sample size was informed by statistics: a sample of at least 384 is needed for 95% confidence, and a 5% margin of error for a population of more than 1,000,000 is needed. The inclusion criteria for participation were men or women from all races and ethnic groups aged 15 years and older, and the exclusion criteria were people under 15 years of age and people unable to read or write. The sample size was calculated by using registered software Raosoft^®^ SurveyWin^®^ ver. 4.5. Service users waiting to see health practitioners, as well as their accompanying family members, were approached to take part in the study and were briefed about it. This was performed in English, isiZulu, or Setswana, as needed, by the second researcher, who can speak all three languages. In the experience of the second researcher, these three languages are commonly used in the clinics where the study was performed. The participants signed the consent form and were subsequently given the research cases and questionnaire.

### 2.3. Research Tool

The questionnaire used in the study which provided the secondary data was divided into two sections, namely, sections A and B. Section A contained the demographic data, and section B contained the questions used to assess mental health literacy. To assess mental health literacy, the respondents were presented with three fictive clinical case studies that met the DSM-5 criteria for mental depressive disorders, schizophrenia, and generalized anxiety disorders [46]. These disorders were selected because anxiety and mood disorders are common in South Africa. Although the global prevalence of schizophrenia is low, it is nonetheless relatively common. Although this is the case, symptoms of schizophrenia seem to be more likely to be identified as mental disorders, especially in African countries [13,14]. Hence, schizophrenia was included. This study asked participants to choose one answer among various options for three questions about the fictive cases: (1) What type of illness do you think the person is suffering from? (2) What do you think caused the persons’ suffering? (See Table 1: Questions about the cases). Thus, following the work of Jorm et al. and Jorm, two specific aspects of mental health literacy were targeted [34,35]. Four items were included as possible options reflecting type of illness and cause of illness. A score 0 zero was captured for an incorrect answer, while a score of 1 was capture for a correct type of illness displayed in the clinical pictures and also for correct cause of mental illness.

### 2.4. Data Analysis

Frequency tables (counts and percentages) were constructed for demographic variables (age, sex, marital status, level of education, area of the clinic, and employment status). Pearson’s chi-square test was performed for participants who chose the answer for recognition and the cause of their mental disorders. To determine the predictors of recognition and causes of mental disorders, hierarchical logistic regression consisting of three levels was used. level 1 included demographic variables (age, sex, marital status, and level of education), level 2 included geographic location, and level 3 included the employment status of the participants. A *p* value less than 0.05 was considered statistically significant.

### 2.5. Logistic Regression

To select the best model among the group of competing models, the null hypothesis testing approach using the likelihood ratio test and the information theoretic model comparison approaches (Akaike information criterion (AIC) and Bayesian information criterion (BIC)) were used. The model with the smallest AIC, BIC, and significant likelihood ratio (LR) is considered the model that best fits the data.

### 2.6. Likelihood Ratio Test

The likelihood-ratio test (LRT) (often called the likelihood-ratio chi-square test) is a null hypothesis test that helps one choose the “best” model between two nested models [48,49]. Nested models mean that one is a special case of the other. The likelihood ratio test is expressed as the ratio between the log-likelihood of a simpler model and a model with more parameters.

### 2.7. Akaike Information Criterion

The AIC is an estimator of prediction error and thereby the relative quality of statistical models for a given set of data, which is useful for comparing model fit and complexity [50,51].

### 2.8. Bayesian Information Criterion

The BIC is a statistical method for model selection among a finite set of models, which favors models that are a good fit for the data while penalizing for complexity [51].

### 2.9. Ethical Considerations

The study received approval from the Tshwane District Research Committee and Research Ethics Committee of the Faculty of Health Sciences, University of Pretoria. Ethics Reference No.: 543/2019. All participants provided consent to participate in the study, and their information was kept anonymous. The participants were informed about the purpose of the study and that they could withdraw at any time without providing any reason. To ensure adherence to privacy and Protection of Personal Information Act (POPI Act), names and images of participants were excluded from the questionnaire, and filling of the questionnaire took place at a designated room at respective local clinics for participants who consented.

## 3. Results

The study included 385 participants with a mean age of 37.39 ± 11.14 years (range: 13–80); additionally, 299 (77.66%) were females, and 86 (22.34%) were males. More than half of the participants were aged 35 years and above, and over 60% of the participants were single, followed by 111 (29.13%) who were married. Approximately 171 (45.12%) and 169 (44.59%) had completed secondary and tertiary education, respectively. Notably, more than 57% of the participants were unemployed, whereas 307 (79.74%) resided in township areas. Table 2 summarizes the participants’ demographic and socioeconomic profiles.

### 3.1. Sociodemographic, Geographic Location and Socioeconomic Correlates of Recognition and Causes of Mental Health Disorders

With respect to the recognition of mental health disorders, there was a positive correlation between the location of the clinic and the recognition of mental health disorders ($\chi^{2}\left(1\right)$: 3.9782; *p* < 0.05), and participants attending clinics in urban areas consistently outperformed those in township areas in identifying correct mental health disorders depicted in the clinical pictures. Furthermore, variables such as sex and the location of the clinic were also positively associated with causes of mental health disorders. Great disparities persisted among males and females ($\chi^{2}\left(1\right):7.7314$; *p* < 0.05), with females consistently outperforming males in the correct identification of mental health disorders. Moreover, urbanicity emerged as a significant correlate of the correct identification of causes of mental health disorders associated with clinical findings ($\chi^{2}\left(1\right):6.5641$; *p* < 0.05). Table 3 summarizes the correlates of recognition and causes of mental health disorders among urban and township clinic attendees.

### 3.2. Sociodemographic, Geographic Location and Socioeconomic Predictors of Recognition of Mental Disorders

The findings show that urban location emerged as a significant predictor of correct recognition of mental health disorders in level 2. Compared with those attending clinics in urban locations, participants attending clinics in township areas had 32% lower odds [OR: 0.32; 95% CI: (0.11, 0.93); Wald $\chi^{2}\left(1\right):4.3681$; *p* value: 0.036] of recognizing correct mental health disorders shown in clinical pictures. Interestingly, participants with primary education were three times more likely to recognize correct mental disorders, although the results were not statistically significant (*p* > 0.05). Table 4 summarizes the regression analysis of socioeconomic and demographic predictors of recognition of mental disorders.

### 3.3. Sociodemographic, Geographic Location and Socioeconomic Predictors of Causes of Mental Disorders

In level 1 (demographic variables), gender emerged as a significant predictor of the correct identification of mental health disorders, particularly females exhibited low levels of knowledge of cause of mental disorders [OR: 0.42; 95% CI: (0.24, 0.72); Wald $\chi^{2}\left(1\right):$ 9.9856; *p* value: 0.002]. In level 2, the addition of geographic location improved the model significance; moreover, gender [OR: 0.41; 95% CI: (0.24, 0.71); Wald $\chi^{2}\left(1\right):\;10.0489$; *p* value: 0.001] and location [OR: 0.42; 95% CI: (0.21, 0.83); Wald $\chi^{2}\left(1\right):6.1504$; *p* value: 0.013] significantly influenced the ability of participants to correctly recognize the cause of mental disorders, participants in township areas could not correctly identify correct cause of mental disorders. Table 5 summarizes the sociodemographic, geographic location, and socioeconomic predictors of the causes of mental disorders.

## 4. Discussion

Due to the non-probability sampling choice of the study, caution should be taken when interpreting the findings of this study. The study underpinned location as a significant predictor of correct recognition of mental health disorder and gender, level of education and location as significant predictors of correct cause of mental health disorder. The results are thematically discussed below.

### 4.1. Recognition of Mental Disorders

The results showed that the location of the clinic was a significant predictor of correct recognition of mental disorders. Participants who attended clinics in urban areas consistently demonstrated greater accuracy in recognizing mental disorders than did those in township or nonurban areas did, a finding that is consistent with several other studies [32,52,53]. This finding may indicate that urbanicity is associated mostly with access to adequate support and resources, including mental health services and resources. Moreover, nurses in township clinics often receive less training and support than their urban counterparts do [54,55]. In China, a more industrialized context, nurses undergo monthly mental health training sessions lasting 45–90 min [56]. In contrast, Khairunnisa et al. caution that urbanicity alone does not confer protection against the development of mental health disorders, suggesting that other sociocultural factors may play a role [53]. The reason for this difference could be attributed to cross-cultural differences in parenting and community stigma concerning mental disorders.

Improving the recognition of mental disorders among township clinic attendees requires a multifaceted approach. This includes targeted training and continuous support for registered nurses, the development and dissemination of culturally appropriate educational resources, and community-based awareness campaigns focused on mental health literacy and disorder-specific identification. Moreover, ensuring equitable access to these resources across urban and township clinics is essential for reducing disparities in mental health literacy. These interventions could be embedded within national health strategies to strengthen primary healthcare and promote help seeking among participants.

### 4.2. Causes of Mental Disorders

Factors such as sex, education and the location of the clinic were significantly associated with the cause of mental disorders. Compared with males, females exhibited a greater ability to recognize correct causes of mental health disorders. This finding was reported by several authors, who also reported gendered disparities, particularly that females continually outperform males in recognizing the correct causes of mental disorders [4,14,33,47]. The reason for this result could be that gender differences in South African mental health literacy are driven by factors such as cultural norms around masculinity, help-seeking, societal pressures, and gender-based violence, which disproportionately affect women and influence how men and women recognize and address mental disorders. Moreover, while females often report greater distress and are more inclined to seek help, males may be deterred by societal stigma and traditional expectations of toughness, leading to poorer mental health literacy and help-seeking behavior [38,39,40]. In contrast to literature findings highlighting the dominance of females in correctly identifying correct cause of mental disorders, a study in KZN showed that Males were more likely to correctly identify correct symptoms and cause for mental disorders [28].

With respect to level of education, participants who attained tertiary education were consistently more likely to recognize mental disorders than those with primary education. Several studies have also reported that higher education is a protective factor for mental health literacy [4,36,37]. The reason for this finding is that education in South African nonurban areas during apartheid was a segregated system designed to limit Black South Africans to menial labor roles, characterized by the Bantu Education Act of 1953, which created underfunded, inferior schools with inadequate resources, high teacher absenteeism, and a curriculum tailored to service rather than intellectual advancement [41,42,43]. Although apartheid education ideology has been officially left behind, schools are still under de facto segregation. Whites are in private schools, and suburban schools have the most Colored students, while township schools are overwhelmingly Black, and rural schools tend to have Black and Colored students. Furthermore, the location of community healthcare clinics was a significant predictor of the correct cause of mental disorders. The participants who attended clinics in urban areas outperformed those in township areas, a result well documented in several studies [32,37,44]. The reason for this finding is that participants in urban locations have easier access to information and health education than do those in nonurban areas [45].

The findings of the current study converge with previous studies which reported that the City of Tshwane, like the broader South Africa, faces an immense mental health burden, characterized by high prevalence rates of disorders such as general anxiety disorder and mental depressive disorders and inequalities in service accessibility [15,16,17]. The national mental health context which indicates that on average 16% of the South African population is affected by mental health issues, and up to 44% of young adults being reportedly living with some form of mental health disorder [15,18], is a call for concern and key stakeholders should pay attention and prioritize access to mental health services. Also, another major health crisis facing the City of Tshwane and South African health system is the treatment gap, with reports indicating that nearly 75% of people with mental health disorders in South Africa do not have access to any form of mental healthcare [16,17]. This is due to a shortage of professionals, limited resources, and uneven distribution of services as a consequence of department of health budget cuts [17,19]. The City of Tshwane has been progressive in the fight for access to mental health services by integrating mental health services into its primary healthcare system and provide various public and private support options [20].

### 4.3. Implications for Policy and Practice

In South Africa’s post-apartheid landscape, persistent disparities in education, healthcare access, and infrastructure continue to shape mental health literacy outcomes. This study highlights the urgent need for community-level policies that prioritize mental health literacy in nonurban settings, where a historical lack of investment has left clinics under resourced, and populations underserved. Culturally responsive mental health education, which is delivered in local languages and embedded within primary healthcare services, can bridge knowledge disparities, and reduce stigma, particularly among males. Training community health workers and nurses in nonurban clinics to recognize and address mental disorders is essential. Moreover, gender-sensitive community outreach strategies should be implemented to address the unique barriers men face in help-seeking, shaped by entrenched norms around masculinity. These interventions should be locally driven, supported by national policy, and aligned with South Africa’s broader goals of equitable health access and social justice.

### 4.4. Limitations of the Study

While this study provides valuable insights into the influence of sociodemographic and geographic location on communities’ MHL, it is important to acknowledge its limitations. Firstly, Due to financial constraints and time limitations, the sample did not include all other clinics within Tshwane Region 1 and other regions in the City of Tshwane. Secondly, the results are not likely generalizable and should therefore be interpreted with caution as they include randomly sampled clinics from region 1. Further research is warranted to extend the study for generalizability of the study findings in the city. Lastly, the study focused only on three mental illnesses, targeted very specific aspects of mental health literacy and sourced participants from very specific areas in the City of Tshwane. Moreover, performing the study at clinics may have selected participants with biases that may be different from people who never use or need to use medical clinics.

## 5. Conclusions

Mental health literacy in South Africa remains deeply shaped by sociodemographic and geographic divides. This study reveals that township communities, which have long been marginalized by educational policies and ongoing structural neglect, continue to face significant barriers in recognizing and understanding mental disorders. Addressing these disparities requires more than clinical interventions and community-driven, culturally grounded strategies that empower local health workers, dismantle stigma, and promote inclusive mental health education. Bridging the urban–township divide is not only a public health imperative but also a step toward realizing the constitutional promise of equitable healthcare for all South Africans. Consequently, due to the use of convenience sampling, the results are not likely generalizable and should therefore be interpreted with caution. Further research is warranted to confirm the findings of the study. Despite the aforementioned limitations, these results offer a meaningful foundation for understanding MHL among secondary school learners and can inform future research and intervention strategies aimed at improving mental health outcomes in similar educational and social contexts. The study recommends that the City of Tshwane should initiate longitudinal and intervention studies to track changes in the MHL over time, especially after community programs and policy interventions. Future studies should assess the cost effectiveness of embedding MHL programs into primary healthcare services.

## Figures and Tables

**Table 1:** Questions about the clinical cases. Source: Madlala et al. [47].

1. What type of illness do you think the person is suffering from?
(a) Physical illness
(b) Mental illness *
(c) None of the above
(d) I do not know
2. What do you think causes the persons’ suffering?
(a) Stress
(b) Punishment from God/ancestors/bewitchment
(c) Brain/genetic/psychological *
(d) None of the above

* Represent the correct response.

**Table 2:** Summary of participants’ socioeconomic and demographic factors.

Variables	*n* (%)
Age	
<18	2 (0.52)
18–35	184 (47.49)
>35	199 (51.69)
Gender	
Female	299 (77.66)
Male	86 (22.34)
Location	
Township	307 (79.74)
Urban	78 (20.26)
Marital status	
Single	246 (65.57)
Married	111 (29.13)
Divorced	12 (3.15)
Widowed	12 (3.15)
Level of education	
Never schooled	8 (2.11)
Special school	6 (1.58)
Primary	25 (6.60)
Secondary	171 (45.12)
Tertiary	169 (44.59)
Employment	
Employed	155 (42.12)
Unemployed	213 (57.88)

**Table 3:** Summary of socioeconomic and demographic correlates of recognition and cause of mental disorders.

Variables	Recognition		*p* Value	$\chi^{2}$	Cause		*p* Value	$\chi^{2}$
	Yes (*n* = 334)	No (*n* = 51)			Yes (*n* = 264)	No (*n* = 116)		
Age								
<18	2 (100%)	0 (0%)	0.646	0.8753	2 (100%)	0 (0%)	0.502	1.3786
18–35	157 (85.33%)	27 (14.67%)			123 (67.58%)	59 (32.42%)		
>35	175 (87.94%)	24 (12.06%)			139 (70.92%)	57 (29.08%)		
Gender								
Female	259 (86.62%)	40 (13.38%)	0.887	0.0202	216 (72.97%)	80 (27.03%)	0.005 *	7.7314
Male	75 (87.21%)	11 (12.79%)			48 (57.14%)	36 (42.86%)		
Location								
Township	261 (85.02%)	46 (14.98%)	0.046 *	3.9782	202 (66.45%)	102 (33.55%)	0.010 *	6.5641
Urban	73 (93.59%)	5 (6.41%)			62 (81.58%)	14 (18.42%)		
Marital status								
Single	212 (86.18%)	34 (13.82%)	0.520	2.2603	169 (69.55%)	74 (30.45%)	0.596	1.8888
Married	95 (85.59%)	16 (14.41%)			74 (67.89%)	35 (32.11%)		
Divorced	11 (91.67%)	1 (8.33%)			10 (83.33%)	2 (16.67%)		
Widowed	12 (100%)	0 (0%)			7 (58.33%)	5 (41.67%)		
Level of education								
Never schooled	8 (100%)	0 (0%)	0.351	4.4267	8 (100%)	0 (0%)	0.088	8.0957
Special school	6 (100%)	0 (0%)			3 (50%)	3 (50%)		
Primary	24 (96%)	1 (4%)			13 (52%)	12 (48%)		
Secondary	146 (85.38%)	25 (14.62%)			118 (69.82%)	51 (30.18%)		
Tertiary	145 (85.80%)	24 (14.20%)			116 (69.88%)	50 (30.12%)		
Employment								
Employed	134 (86.45%)	21 (13.55%)	0.953	0.0003	106 (69.28%)	47 (30.72%)	0.8268	0.0578
Unemployed	184 (86.38%)	29 (13.62%)			143 (68.10%)	67 (31.90%)		

* Statistical significance *p* < 0.05.

**Table 4:** Hierarchical logistic regression analysis of socioeconomic and demographic predictors of recognition of mental disorders.

Predictors	Level 1 (Demographics)			Level 2 (Geographic Location)			Level 3 (Employment Status)		
	OR (95%CI)	*p* Value	Wald *χ*^2^ Statistics	OR (95%CI)	*p* Value	Wald *χ*^2^ Statistics	OR (95%CI)	*p* Value	Wald *χ*^2^ Statistics
Age									
>35	1	1		1	1		1	1	
18–35	0.78 (0.41, 1.50)	0.458	0.5476	0.77 (0.40, 1.49)	0.441	0.5929	0.77 (0.40, 1.49)	0.440	0.5929
Gender									
Female	1	1		1	1		1	1	
Male	0.95 (0.45, 2.00)	0.900	0.0169	0.95 (0.45, 2.00)	0.886	0.0196	0.95 (0.45, 2.06)	0.899	0.0169
Marital status									
Married	1	1		1	1		1	1	
Single	0.99 (0.51, 1.93)	0.982	0.0004	1.21 (0.61, 2.40)	0.592	0.2916	1.21 (0.61, 2.40)	0.593	0.2809
Level of education									
Tertiary	1	1		1	1		1	1	
Primary	3.21 (0.41, 25.35)	0.268	1.2321	3.24 (0.41, 25.65)	0.266	1.2321	3.22 (0.40, 25.83)	0.270	1.21
Secondary	0.93 (0.50, 1.73)	0.827	0.0484	0.94 (0.51, 1.75)	0.847	0.0361	0.94 (0.50, 1.76)	0.844	0.04
Location									
Urban	1	1		1	1		1	1	
Township				0.32 (0.11, 0.93)	0.036 *	4.3681	0.31 (0.11, 0.93)	0.037 *	4.3681
Employment									
Employed	1	1		1	1		1	1	
Unemployed							1.01 (0.53, 1.96)	0.966	0.0016

* Statistical significance *p* < 0.05.

**Table 5:** Hierarchical logistic regression analysis of socioeconomic and demographic predictors of the cause of mental disorders.

Predictors	Level 1 (Demographics)			Level 2 (Geographic Location)			Level 3 (Employment Status)		
	OR (95%CI)	*p* Value	Wald *χ*^2^ Statistics	OR (95%CI)	*p* Value	Wald *χ*^2^ Statistics	OR (95%CI)	*p* Value	Wald *χ*^2^ Statistics
Age									
>35	1	1		1	1		1	1	
18–35	0.62 (0.37, 1.04)	0.070	3.1684	0.62 (0.37, 1.04)	0.068	3.2041	0.62 (0.37, 1.04)	0.070	3.1684
Gender									
Female	1	1		1	1		1	1	
Male	0.40 (0.23, 0.71)	0.002 *	9.9856	0.41 (0.24, 0.71)	0.001 *	10.0489	0.40 (0.23, 0.71)	0.002 *	9.7969
Marital status									
Married	1	1		1	1		1	1	
Single	1.18 (0.70, 1.99)	0.529	0.3969	1.42 (0.83, 2.46)	0.202	1.6129	1.43 (0.83, 2.47)	0.198	1.6641
Divorced	2.20 (0.43, 11.28)	0.346	0.9025	1.94 (0.38, 9.89)	0.426	0.64	1.95 (0.38, 9.96)	0.422	0.68
Widowed	0.33 (0.09, 1.30)	0.115	2.4649	0.33 (0.08, 1.33)	0.119	2.4025	0.33 (0.08, 1.35)	0.123	2.3716
Level of education									
Tertiary	1	1		1	1		1	1	
Primary	0.29 (0.11, 0.75)	0.010 *	6.5536	0.29 (0.11, 0.74)	0.010 *	6.5536	0.29 (0.11, 0.77)	0.013 *	6.1504
Secondary	0.92 (0.56, 1.49)	0.724	0.1156	0.92 (0.56, 1.49)	0.723	0.1225	0.92 (0.56, 1.51)	0.751	0.0961
Special school	0.71 (0.10, 4.81)	0.726	0.1225	0.84 (0.12, 5.81)	0.857	0.0324	0.82 (0.12, 5.76)	0.843	0.0361
Location									
Urban	1	1		1	1		1	1	
Township				0.42 (0.21, 0.83)	0.013 *	6.1504	0.43 (0.21, 0.85)	0.016 *	5.8081
Employment									
Employed	1	1		1	1		1	1	
Unemployed							0.93 (0.56, 1.54)	0.764	0.09

* Statistical significance *p* < 0.05.

## Data Availability

The raw data that supported the findings of this study are available from the corresponding author, O.P.M., upon fair and reasonable request.

## Abbreviations

The following abbreviations are used in this manuscript:

AIC	Akaike information criterion
BIC	Bayesian information criterion
LR	Likelihood ratio
HLR	Hierarchical logistic regression
LRT	Likelihood ratio test
MHL	Mental health literacy

## References

[1] Jarso M.H., Debele G.R., Gezimu W., Nigatu D., Mohammedhussein M., Mamo A., Dule A., Hassen M., Jemal K. (2022). Knowledge, attitude, and its correlates of the community toward mental illness in Mattu, South West Ethiopia. Front. Psychiatry.

[2] Al-Qerem W., Jarab A., Khdour M., Eberhardt J., Alasmari F., Hammad A., Zumot R., Khalil S. (2024). Assessing mental health literacy in Jordan: A factor analysis and Rasch analysis study. Front. Public Health.

[3] Solhi M., Saboohi Z., Nazarnia M., Gudarzi F., Roodaki L., Nouri R. (2024). Mental health literacy and its relationship with positive mental health in Iranian adolescents. Int. J. Sch. Health.

[4] Chu Z., Xie J., Liu R., Ou L., Lan Z., Xie G. (2025). Gender-specific differences in mental health literacy and influencing factors among residents in Foshan City, China: A cross-sectional study. Front. Public Health.

[5] Schillinger D. (2021). Social determinants, health literacy, and disparities: Intersections and controversies. HLRP Health Lit. Res. Pract..

[6] Amoah P.A., Ameyaw E.K., Fordjour G.A. (2024). Interplay of sociocultural factors, health literacy and well-being among African asylum seekers and refugees in Asia: A systematic review. J. Migr. Health.

[7] Kirkbride J.B., Anglin D.M., Colman I., Dykxhoorn J., Jones P.B., Patalay P., Pitman A., Soneson E., Steare T., Wright T. (2024). The social determinants of mental health and disorder: Evidence, prevention and recommendations. World Psychiatry.

[8] de Villiers K. (2021). Bridging the health inequality gap: An examination of South Africa’s social innovation in health landscape. Infect. Dis. Poverty.

[9] Bell G.J., Ncayiyana J., Sholomon A., Goel V., Zuma K., Emch M. (2022). Race, place, and HIV: The legacies of apartheid and racist policy in South Africa. Soc. Sci. Med..

[10] Muyambi G.C., Ahiaku P.K.A. (2025). Inequalities and education in South Africa: A scoping review. Int. J. Educ. Res. Open.

[11] Herman A.A., Stein D.J., Seedat S., Heeringa S.G., Moomal H., Williams D.R. (2009). The South African Stress and Health (SASH) study: 12-month and lifetime prevalence of common mental disorders. S. Afr. Med. J..

[12] American Psychiatric Association (2013). Diagnostic Statistical Manual of Mental Disorders.

[13] Atilola O. (2015). Level of community mental health literacy in sub-Saharan Africa: Current studies are limited in number, scope, spread, and cognizance of cultural nuances. Nord. J. Psychiatry.

[14] Deribew A., Tamirat Y.S. (2021). How are mental health problems perceived by a community in Agaro town?. Ethiop. J. Health Dev..

[15] Bantjes J., Lochner C., Saal W., Roos J., Taljaard L., Page D., Auerbach R.P., Mortier P., Bruffaerts R., Kessler R.C. (2019). Prevalence and sociodemographic correlates of common mental disorders among first-year university students in post-apartheid South Africa: Implications for a public mental health approach to student wellness. BMC Public Health.

[16] South African College of Applied Psychology (2019). The Shocking State of Mental Health in South Africa in 2019. https://www.sacap.edu.za/blog/management-leadership/mental-health-south-africa/.

[17] Sorsdahl K., Petersen I., Myers B., Zingela Z., Lund C., Van der Westhuizen C. (2023). A reflection of the current status of the mental healthcare system in South Africa. SSM-Ment. Health.

[18] National Planning Commission (2024). Mental Health Situational Analysis: South Africa. https://www.nationalplanningcommission.org.za/assets/Documents/Mental%20Health%20Situational%20Analysis%20South%20Africa%20final%20Report_May%202024.pdf.

[19] Ndlovu N., Blose N., Mokganya M. (2025). Health and related indicators: A mental health focus. S. Afr. Health Rev..

[20] Ramadie O. (2024). City of Tshwane Introduces Mental Health Services at Its 24 Clinics. https://www.tshwane.gov.za/?p=75172.

[21] Yu Y., Liu Z.-W., Hu M., Liu X.-G., Liu H.-M., Yang J.P., Zhou L., Xiao S.-Y. (2015). Assessment of mental health literacy using a multifaceted measure among a Chinese rural population. BMJ Open.

[22] Aluh D.O., Anyachebelu O.C., Anosike C., Anizoba E.L. (2018). Mental health literacy: What do Nigerian adolescents know about depression?. Int. J. Ment. Health Syst..

[23] Aluh D.O., Okonta M.J., Odili V.U. (2019). Cross-sectional survey of mental health literacy among undergraduate students of the University of Nigeria. BMJ Open.

[24] Mideksa G., Tesfaye E., Yitayih Y., Sime Y., Aliye K., Gizaw A.T. (2024). Mental health literacy and associated factors among traditional healers of Jimma town, southwest, Ethiopia 2020: A community based, cross-sectional study. Front. Psychiatry.

[25] Zita C.T. (2018). Mental Health Literacy of Young People in South Africa: A Study of University of Kwazulu-Natal Students. Doctoral Dissertation.

[26] Madlala D.P., Joubert P., Mokoena O.P. (2025). Mental health literacy among secondary school learners in Tshwane region 1: A quantitative study. S. Afr. J. Psychiatry.

[27] Matsoele N.F.C., Tadi N.F. (2024). Mental health literacy in black South African communities. J. Psychol. Afr..

[28] Nzama N.S.K. (2020). Mental Health Literacy: Conceptions and Attitudes Towards Depression and Schizophrenia Among African Residents of the eThekwini District Municipality. Doctoral Dissertation.

[29] Smit C.A., Marais B.S. (2025). Assessing the mental health literacy of healthcare workers at a Johannesburg tertiary hospital. S. Afr. J. Psychiatry.

[30] Kim Y., Choi Y.K., Emery S. (2013). Logistic regression with multiple random effects: A simulation study of estimation methods and statistical packages. Am. Stat..

[31] Tesfaye Y., Agenagnew L., Anand S., Tucho G.T., Birhanu Z., Ahmed G., Getnet M., Yitbarek K. (2021). Knowledge of the community regarding mental health problems: A cross-sectional study. BMC Psychol..

[32] Nishio A., Marutani T. (2022). Mental health literacy survey among Cambodia’s urban and rural populations: Results from a vignette-based population survey. PLoS ONE.

[33] He X.Y., Tan W.Y., Guo L.L., Ji Y.Y., Jia F.J., Wang S.B. (2024). Mental health literacy among urban and rural residents of Guangdong province, China. Risk Manag. Healthc. Policy.

[34] Jorm A.F., Korten A.E., Jacomb P.A., Christensen H., Rodgers B., Pollitt P. (1997). “Mental health literacy”: A survey of the public’s ability to recognize mental disorders and their beliefs about the effectiveness of treatment. Med. J. Aust..

[35] Jorm A.F. (2000). Mental health literacy: Public knowledge and beliefs about mental disorders. Br. J. Psychiatry.

[36] Hadjimina E., Furnham A. (2017). Influence of age and gender on mental health literacy of anxiety disorders. Psychiatry Res..

[37] Shi P., Yang A., Zhao Q., Chen Z., Ren X., Dai Q. (2021). A hypothesis of gender differences in self-reporting symptom of depression: Implications to solve underdiagnosis and undertreatment of depression in males. Front. Psychiatry.

[38] Bam C. (2024). Male Adolescent Masculinity and Help-Seeking Behavior: A Qualitative Study in Stellenbosch, Western Cape. Doctoral Dissertation.

[39] Johnson A., Amonoo L., Lofton S., Powell-Roach K.L. (2024). How masculinity impedes African American men from seeking mental health treatment. Am. J. Men’s Health.

[40] Mokhwelepa L.W., Sumbane G. (2025). Men’s Mental Health Matters: The Impact of Traditional Masculinity Norms on Men’s Willingness to Seek Mental Health Support; a Systematic Review of Literature. Am. J. Men’s Health.

[41] Ocampo M.L. (2004). A Brief History of Educational Inequality from Apartheid to the Present. Global Perspectives on Human Language: The South African Context. https://web.stanford.edu/~jbaugh/saw/Lizet_Education_Inequity.html.

[42] South African History Online (2013). The June 16 Soweto Youth Uprising. South African History Online. https://sahistory.org.za/article/june-16-soweto-youth-uprising.

[43] Gallo M.A. (2020). Bantu Education, and Its Living Educational and Socioeconomic Legacy in Apartheid and Postapartheid South Africa. https://research.library.fordham.edu/international_senior/43.

[44] Mwambwa-Johnson E.Y. (2021). Mental Health Literacy Among Rural and Urban Young Adults in Zambia. Doctoral Dissertation.

[45] Mumbauer A.E., Stein D.J., Wolvaardt G.G. (2025). Targeting youth mental health in a demographically young country: A scoping review focused on South Africa. Int. Rev. Psychiatry.

[46] Regier D.A., Kuhl E.A., Kupfer D.J. (2013). The DSM-5: Classification and criteria changes. World Psychiatry.

[47] Madlala D., Joubert P.M., Masenge A. (2022). Community mental health literacy in Tshwane region 1: A quantitative study. S. Afr. J. Psychiatry.

[48] Lewis F., Butler A., Gilbert L. (2011). A unified approach to model selection using the likelihood ratio test. Methods Ecol. Evol..

[49] Chen Y., Moustaki I., Zhang H. (2020). A note on likelihood ratio tests for models with latent variables. Psychometrika.

[50] Akaike H. (1987). Factor analysis and AIC. Psychometrika.

[51] Chakrabarti A., Ghosh J.K. (2011). AIC, BIC and recent advances in model selection. Philosophy of Statistics.

[52] Schroeder S., Tan C.M., Urlacher B., Heitkamp T. (2021). The role of rural and urban geography and gender in community stigma around mental illness. Health Educ. Behav..

[53] Khairunnisa M., Yunitawati D., Latifah L., Effendi D.E., Fitrianti Y., Handayani S., Kusumawardani H.D. (2024). Rural-urban differences in common mental disorders among Indonesian youth: A cross-sectional national survey. Osong Public Health Res. Perspect..

[54] Mutshatshi T.E., Mothiba T.M., Malema R.N. (2022). Exploration of in-service training needs for nurses implementing the nursing process at regional hospitals of Limpopo Province, South Africa. Open Public Health J..

[55] Siyothula E.T.B. (2022). Experiences and views of clinical psychologists working in nonurban areas of KwaZulu-Natal, South Africa. S. Afr. J. Psychol..

[56] Tan X., Pan M., Wan Z., Yang Y., Zhang L., Fang Y., Li X., Shen M. (2024). Current status and needs of in-service training for psychiatric nurses in 24 provinces of China: A cross-sectional survey. Front. Psychol..

